# [Corrigendum] Resveratrol reverses P‑glycoprotein‑mediated multidrug resistance of U2OS/ADR cells by suppressing the activation of the NF‑κB and p38 MAPK signaling pathways

**DOI:** 10.3892/ol.2024.14225

**Published:** 2024-01-10

**Authors:** Rui Zhang, Ming Lu, Zhen Zhang, Xiliang Tian, Shouyu Wang, Decheng Lv

Oncol Lett 12: 4147–4154, 2016; DOI: 10.3892/ol.2016.5136

Subsequently to the publication of the above article, an interested reader drew to the authors’ attention that the β-actin bands shown in Figs. 5D and 6 appeared to be striking similar, albeit that one set of the protein bands were featured in the opposite orientation relative to the other. The authors have consulted their original data, and realized that the western blots were incorporated incorrectly into Fig. 5D.

The corrected version of Fig. 5, showing the correct control blots for Fig. 5D, is shown below. Note that the errors in this figure did not affect either the results or the conclusions reported in this study. The authors are grateful to the Editor of *Oncology Letters* for granting them the opportunity to publish this corrigendum, and regret any inconvenience caused to the readership of the Journal.

## Figures and Tables

**Figure 5.**
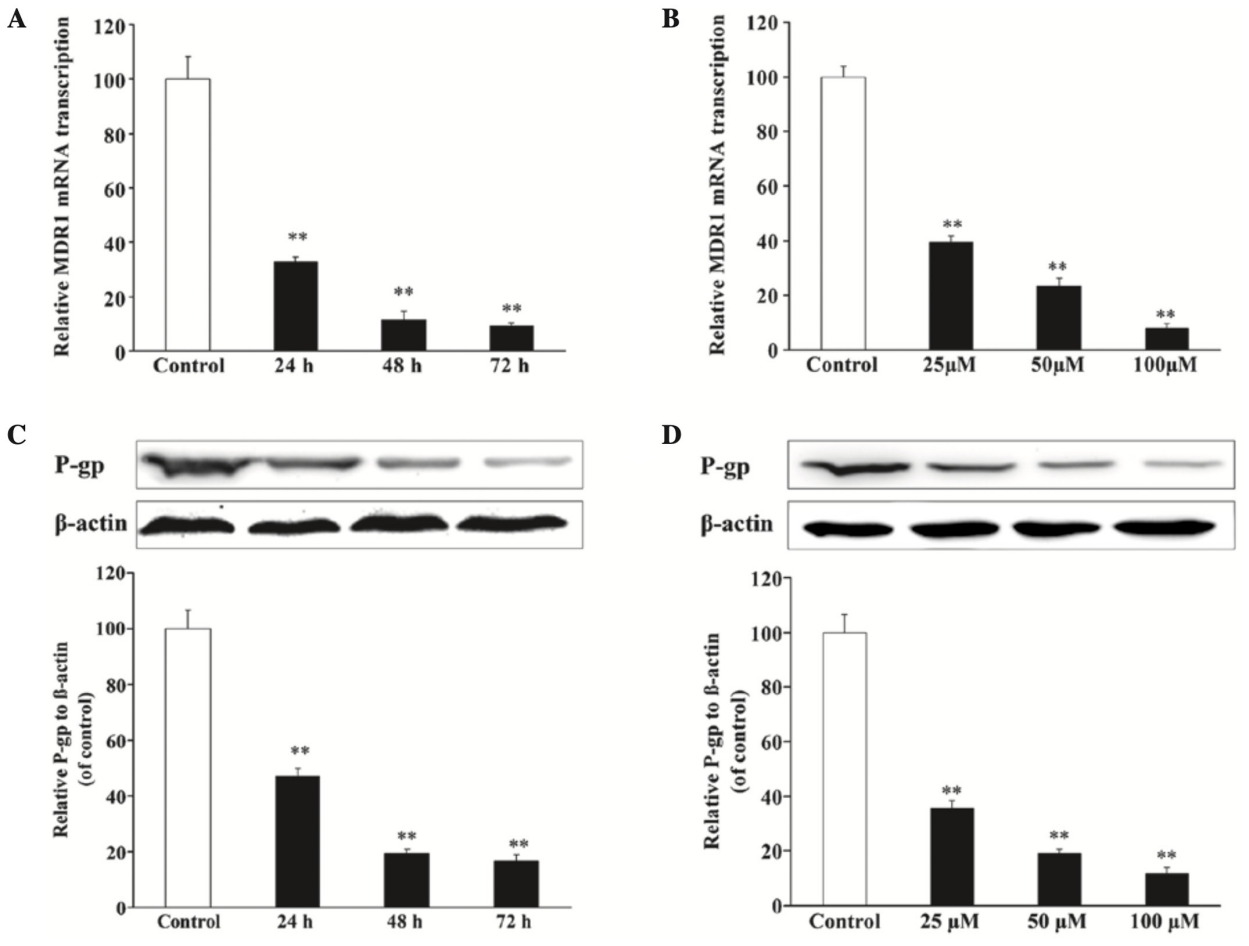
Effects of resveratrol on MDR1/P-gp expression in U2OS/adriamycin cells. Cells were incubated with (A) 100 µM resveratrol for 0–72 h or (B) with various concentrations of resveratrol for 48 h. MDR1 messenger RNA expression was analyzed by reverse transcription-quantitative polymerase chain reaction. Cells were incubated with (C) 100 µM resveratrol for 0–72 h or (D) with various concentrations of resveratrol for 48 h. P-gp expression was analyzed by western blotting and normalized to β-actin level. Data are expressed as the mean ± standard deviation (**P<0.01 vs. untreated U2OS cells group; n=6). P-gp, P-glycoprotein; mRNA, messenger RNA; MDR1, multidrug resistance protein 1.

